# MMP‐2 and MMP‐13 affect vasculogenic mimicry formation in large cell lung cancer

**DOI:** 10.1111/jcmm.13283

**Published:** 2017-08-02

**Authors:** Yanlei Li, Baocun Sun, Xiulan Zhao, Xudong Wang, Danfang Zhang, Qiang Gu, Tieju Liu

**Affiliations:** ^1^ Department of Pathology Tianjin Medical University Tianjin China; ^2^ Department of Pathology Tianjin Cancer Hospital Tianjin Medical University Tianjin China; ^3^ Department of Pathology Tianjin General Hospital Tianjin Medical University Tianjin China; ^4^ Department of Maxillofacial and Otorhinolaryngology Head and Neck Surgery Tianjin Medical University Cancer Institute and Hospital Tianjin China

**Keywords:** MMP‐2, MMP‐13, large cell lung cancer, vasculogenic mimicry, laminin5

## Abstract

Matrix metalloproteinases (MMPs) have critical functions in tumour vasculogenic mimicry (VM). This study explored the mechanisms underlying MMP‐13 and MMP‐2 regulation of tumour VM formation in large cell lung cancer (LCLC). In our study, laminin5 (Ln‐5) fragments cleaved by MMP‐2 promoted tubular structure formation by the LCLC cell lines H460 and H661 in three‐dimensional (3D) cultures. Transient up‐regulation of MMP‐13 or treatment with recombinant MMP‐13 protein abrogated tubular structure formation of H460 cells in 3D culture. Treated cells with Ln‐5 fragments cleaved by MMP‐2 stimulated EGFR and F‐actin expression. Ln‐5 fragments cleaved by MMP‐13 decreased EGFR/F‐actin expression and disrupted VM formation. MMP‐13 expression was negatively correlated with VM, Ln‐5 and EGFR in LCLC tissues and xenograft. *In vivo* experiments revealed that VM was decreased when the number of endothelium‐dependent vessels (EDVs) increased during xenograft tumour growth, whereas MMP‐13 expression was progressively increased. In conclusion, MMP‐2 promoted and MMP‐13 disrupted VM formation in LCLC by cleaving Ln‐5 to influence EGFR signal activation. MMP‐13 may regulate VM and EDV formation.

## Introduction

Large cell lung carcinoma is the least common but the most serious type of non‐small cell lung cancer (NSCLC). Rapid growth and early lymph and blood metastasis in NSCLC can lead to a poor prognosis. Tumour growth and metastasis depend on effective microcirculation. There are three microcirculation patterns that can be found in tumours: EDVs, VM and mosaic vessels that are lined by endothelial and tumour cells 1, 2. The VM concept was introduced by Maniotis in 1999 to describe the unique ability of highly aggressive tumour cells to form capillary‐like and extracellular matrix (ECM)‐rich tubular networks without the participation of endothelial cells, and these networks were reported to be associated with metastasis and reduced survival times in patients with tumours 3. Morphological characterization showed that VM networks are rich in laminins and lined by tumour cells. Tumours with aggressive VM formation have high expression levels of the basement membrane ECM component laminin5γ2 and metalloproteinases (MMPs) 4. The increased expression and specific localization of these ECM components suggest that highly aggressive tumour cells can modify their ECM to initiate or promote VM. The laminin5γ2 chain can be cleaved by activated MMP‐2 and MT1‐MMP, thereby producing the cleaved fragments γ2 and γ2x. These fragments are implicated in tumour cell invasion and metastasis and initiate or promote VM network formation in 3D culture 5, 6. However, the mechanism by which these fragments trigger VM formation in tumour cells remains unknown. Evidence has demonstrated that processing of the rat Ln‐5 γ2 chain releases an internal fragment containing three of the four EGF‐like motifs in domain III (DIII), which binds the EGF receptor (EGFR) and induces its phosphorylation 7. Researchers have found that EGFR is linked to the actin cytoskeleton in cells, creating a cell signalling microdomain 8. Cell cytoskeletal rearrangement also facilitates tumour cell VM formation, and several genes can enhance the formation of actin stress fibres and membrane ruffles and reduce lamellipodial protrusions, filopodia, tight junctions and adherens junctions to promote aggressive characteristics, such as migration and VM, in cells 9. We hypothesize that cytoskeletal rearrangement through cell surface receptor responses to these fragments is involved in cell VM formation.

Aggressive tumour cells that compose VM tubes showed substantial plasticity in 3D culture. Matrix metalloproteinase‐13 (MMP‐13) is a proteolytic enzyme that belongs to the family of ECM‐degrading endopeptidases that are characterized by a zinc‐binding motif at their catalytic site 10. MMP‐13 has broad substrate specificity, and it was reported to lead to the disorganization and fragmentation of tight junction proteins, enhancing the permeability of endothelial cells 11. Given that MMP‐13 has been shown to play critical roles in endothelium‐dependent angiogenesis, we investigated the role of MMP‐13 in VM formation. Thus far, there have been no data on the role of MMP‐13 in VM. Here, we address the hypothesis that Ln‐5 fragments cleaved by MMP‐2 activate the EGFR and induce cytoskeletal rearrangement. Additionally, we studied the role of MMP‐13‐mediated proteolytic generation of this ECM fragment in VM formation in lung cancer using human LCLC tissue samples and cell lines. Our evidence suggests that MMP‐13 negatively regulates VM formation in LCLC.

## Materials and methods

### Cell culture

The human LCLC cell lines H460 and H661 (Cell Resource Center, Institute of Basic Medical Sciences, Chinese Academy of Medical Sciences, School of Basic Medicine, Peking Union Medical College) were cultured in RPMI‐1640 with 10% foetal bovine serum (Invitrogen, Grand Island, NY, USA).

### Expression plasmids and MMP‐13 gene silencing

Full‐length MMP‐13 complementary DNA (cDNA) was generated with normal human embryo total cDNA, digested with XhoI/EcoRI and subcloned into pcDNA3.1 vectors. The resulting constructs were confirmed *via* DNA sequencing. A small interfering RNA (siRNA) kit (pRNAT‐MMP‐13‐shRNA) was purchased from GeneScript (Piscataway, NJ, USA). The target sequence AACAGCUGCACUUAUCUUCUUAACU was used to down‐regulate human MMP‐13 *in vitro*. A non‐effective shRNA (target sequence 5ʹ‐AATTCTCCGAACGTGTCACGT‐3ʹ) was used as a negative control. Transfection of H460 and H661 cells was performed with Lipofectamine 2000 (Invitrogen, Carlsbad, CA, USA). G418 was used to select clones to establish stable H460 cells that overexpressed MMP‐13 and H661 cells with reduced expression of MMP‐13.

### Primary antibodies used in this study

Antibodies against MMP‐13 (ab39012, Abcam, Cambridge, MA, USA), MMP‐2 (sc‐13595, Santa Cruz), EGFR (sc‐03, Santa Cruz, Dallas, TX, USA), Ln‐5 (ab14509, Abcam), α‐tubulin (ab27074, Abcam), vimentin (2707, Epimics), CD34 (ZA‐0550, Zhongshan Chemical Co., Beijing, China) and endomucin (Zhongshan Chemical Co.) were used in the experiments, and fluorescent phallotoxin (P5282, Sigma‐Aldrich, St. Louis, MO, USA) was used to identify filamentous actin. ERK 1/2 (sc‐514302, Santa Cruz), Raf‐1 (sc‐373722, Santa Cruz) and Akt1/2/3 (sc‐8312, Santa Cruz) were also obtained.

### Western blot analysis

Cell lysate samples were loaded onto SDS–PAGE gels and transferred to polyvinylidene difluoride membranes. The membranes were blocked with 5% non‐fat milk, incubated with primary antibodies as previously described, incubated with horseradish peroxidase (HRP)‐conjugated secondary antibodies and then visualized with enhanced chemiluminescence (ECL) reagents (Amersham Pharmacia) according to the manufacturer's protocol. β‐Actin (ab1801, Abcam) was used as an internal control.

### 3D culture assay

This assay was conducted as previously described 12, 13. Briefly, 96‐well plates were coated with 20 μl of Matrigel, which was allowed to gel (1 hr, 37°C). H460 and H661 cells were seeded in complete RPMI‐1640 onto the gel and incubated at 37°C for 48 hrs. Either exogenous Ln‐5 or Ln‐5 cleaved by MMP‐13 or MMP‐2 at 37°C for 6 hrs was added into the culture medium when seeding the cells (200 ng/ml). Capillary‐like structure formation was filmed under a phase contrast microscope.

### Cleavage of Ln‐5 by MMP‐2 and MMP‐13

Purified Ln‐5 (100 ng; Abcam, ab42326) was treated with 50 nM human active recombinant MMP‐2 (Cat. No. PF023, Millipore, Darmstadt, Germany) or MMP‐13 (Cat. No. 44287, Millipore) and incubated in 50 mM Tris‐HCl (pH 7.5), 10 mM CaCl_2_, 150 mM NaCl, 1 mM ZnCl_2_ and 0.02% NaN_3_ for 6 hrs at 37°C. Then, all reactions were stopped with 5× denaturing buffer [0.15 M Tris (pH 6.7), 6% SDS, 20% glycerol, 0.1 M EDTA and 0.03% bromophenol blue]. The cleavage fragments were separated on SDS‐PAGE gels under reducing conditions and detected with silver staining, and then, the target band was extracted to detect the amino acid sequence using LC‐MS/MS.

### Patients, immunohistochemistry (IHC), CD34/periodic acid Schiff (PAS) double‐staining and immunofluorescence

Tissue specimens were obtained from 51 patients who had undergone surgical resection for LCLC in Tianjin Medical University Cancer Institute from October 1990 to November 2010. The diagnoses of these samples were verified by two pathologists according to the standards of classification. The protocols were approved by the Ethics Committee of Tianjin Medical University for tissue sample collection, and informed consent was obtained from all subjects. The protocols were carried out in accordance with the approved guidelines.

All human LCLC and xenograft paraffin‐embedded tumour tissues were cut at a thickness of 4 μm. Antigen retrieval was accomplished *via* heat retrieval. Endogenous peroxidase activity was blocked using 3% hydrogen peroxide (in fresh methanol) for 15 min. at room temperature. Then, tissue sections were stained with primary antibodies as previously described. HRP‐labelled rabbit antimouse IgG (Dako Envision plus System) was used as a secondary antibody and a FITC‐ or TRITC‐conjugated secondary antibody was used for immunofluorescence staining. Positive staining was visualized with DAB. Tumour cells with cytoplasmic and/or membrane immunohistochemical labelling were considered positive cells. The percentage of positive tumour cells was determined in three separate fields and from at least 1000 adjacent cells in the area with the highest density of positive cells for each slide. The number of positively labelled tumour cells was scored as follows: 0, 0%; 1, 1–10%; 2, 11–33%; 3, 34–66%; and 4, 67–100%. The intensity of staining was also evaluated and graded from 1 to 3, where 1 indicates weak staining; 2, moderate staining; and 3, strong staining. The two values obtained were multiplied to calculate a receptor score (maximum value = 12). For statistical analysis, the samples were grouped into negative (score ≤ 2) or positive (score > 2) groups. Slides were evaluated by two blinded observers.

For PAS staining, the slides were exposed to 0.5% periodic acid solution for 15 min. and then to Schiff solution for 15–30 min. in the dark without being counterstained with haematoxylin. For CD34‐PAS double‐staining, after the CD34 IHC staining was performed, the slides were stained with PAS as described above and then counterstained with haematoxylin.

### Animal studies

A total of 25 4‐week‐old, male BALB/c nude mice (purchased from Beijing HFK Bioscience Co., Ltd., Beijing, China) were divided randomly into two groups: an H460‐control group (20 mice, five mice for control and 15 mice for time‐dependent experiments) and an H460‐MMP‐13 transfection group (five mice). Each mouse was subcutaneously injected in the right armpit with 5 × 10^6^ cells. Tumour size was measured every 3 d with a sliding caliper. Tumour volume was calculated using the following formula: $\text{volume}=(\text{length}\;\left[\text{mm}\right]\;\times \;{\text{width}}^{2}\;\left[\text{mm}\right])/2$ . Mice in the H460‐control group and H460‐MMP‐13 transfection group were killed after 5 weeks. For the H460 time‐dependent experimental group, five mice were killed each week from each group. Tumour samples were fixed in formalin, embedded in paraffin, cut into slide‐mounted sections (4 μm thick), and immunochemically stained. All animal experiments were approved by the Ethics Committee of Tianjin Medical University. The protocols were performed in accordance with the approved guidelines.

### Statistical analysis

All data in the study were evaluated with SPSS17.0 software (SPSS, Chicago, IL, USA). Data are presented as the mean ± S.D. When two groups were compared, Student's *t*‐test was used. A chi square test was performed to determine correlations among the various parameters. Differences were considered significant at a value of *P* ≤ 0.05.

## Results

### Role of MMP‐2 and MMP‐13 in VM formation

**Figure 1:**
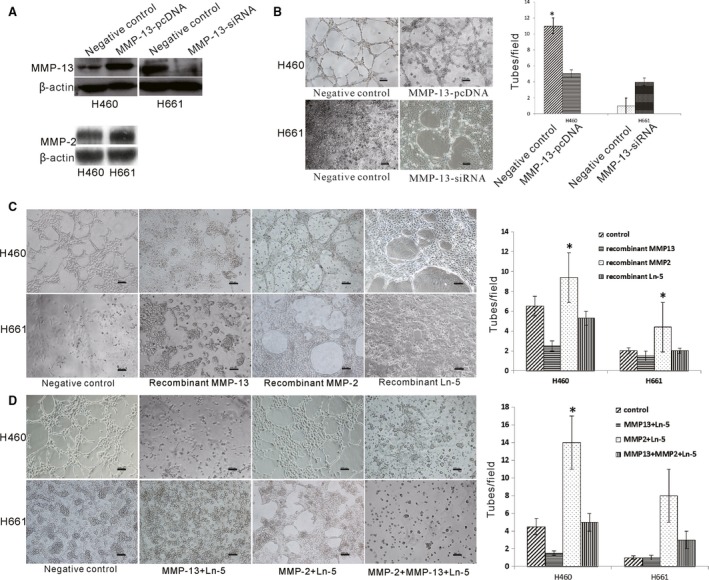
Transfection of cells with MMP‐13 and treatment with MMP‐13 Ln‐5 cleavage fragments disrupted VM formation ( *P < 0.05). (**A**) MMP‐13 expression was successfully regulated by transfection plasmids. In H460 cells, MMP‐13 was up‐regulated by transfection with pcDNA‐MMP‐13 plasmids, and in H661 cells, MMP‐13 was down‐regulated by transfection with MMP‐13‐siRNA plasmids. Transfection efficiency was confirmed *via* WB. MMP‐2 was highly expressed in both cell lines (**B**) Transfection of H460 cells with pcDNA‐MMP‐13 abrogated the capillary‐like tube formation ability on Matrigel. However, knockdown of MMP‐13 expression in H661 cells induced typical tube formation. (**C**) Exogenous addition of recombinant human MMP‐13 proteins in the 3D culture medium resulted in the inability of H460 and H661 cells to form capillary‐like tubes. In contrast, the exogenous addition of recombinant human MMP‐2 proteins induced the cells to form more typical capillary‐like tubes on the Matrigel. Exogenous addition of recombinant human Ln‐5 protein induced the H460 cells to form more typical capillary‐like tubes but not in H661 cells that overexpressed MMP‐13. (**D**) Exogenous addition of Ln‐5 cleaved by MMP‐13 in the 3D culture medium resulted in the inability of H460 and H661 cells to form capillary‐like tubes. In contrast, the exogenous addition of the MMP‐2 Ln‐5 cleavage products induced the cells to form more typical capillary‐like tubes. The addition of all three of these proteins also suppressed tube formation (Bar = 100 μm).

Researchers have found that Ln‐5 cleavage fragments (cleaved by MMP2) are required for VM. To investigate the role of MMP‐13 in VM formation, we studied the effects of MMP‐13 in the human LCLC cell lines H460 and H661. The MMP‐13 expression levels were confirmed *via* Western blotting (WB). H460 cells were then selected for MMP‐13 up‐regulation, and H661 cells were selected for MMP‐13 down‐regulation (Fig. 1A). The expression levels of MMP‐13 were shown to be efficiently up‐regulated by transfection with pcDNA/MMP‐13 and down‐regulated after transfection with MMP‐13 siRNA. We observed that the MMP‐13‐silenced H460 cells could form tubes whereas the MMP‐13‐overexpressing H661 cells could not form tubes in Matrigel. Capillary tube formation was significantly suppressed in H460 cells with up‐regulated MMP‐13 expression, whereas the capillary tube formation ability of H661 cells with down‐regulated MMP‐13 expression was increased (Fig. 1B). In addition, capillary‐like tube formation by H460 or H661 cells on Matrigel was disrupted by treatment with pure MMP‐13, and tube formation was promoted by treatment with pure MMP‐2; there was no significant change after treatment with Ln‐5 (Fig. 1C).

Cleavage of Ln‐5 by MMP‐2 has been reported to induce VM formation. We examined whether the different MMP‐2 and MMP‐13 cleavage products of Ln‐5 play different roles in VM formation. We found that the capillary‐like tube formation by H460 or H661 cells on Matrigel was almost completely abolished after treatment with Ln‐5 cleaved by MMP‐13. Following treatment with exogenous Ln‐5 MMP‐2 cleavage fragments, the cells formed more typical vasculogenic‐like structures. (Fig. 1D). The Ln‐5 fragments cleaved by MMP‐13 and MMP‐2 were more effective in regulating capillary‐like network formation than pure MMP‐13 or MMP‐2. The 3D culture showed that the other Ln‐5 fragments had no effects on VM formation (Fig. S1A).

### The amino acid sequence of the MMP‐13 Ln‐5 cleavage product was detected with LC‐MS/MS

**Figure 2:**
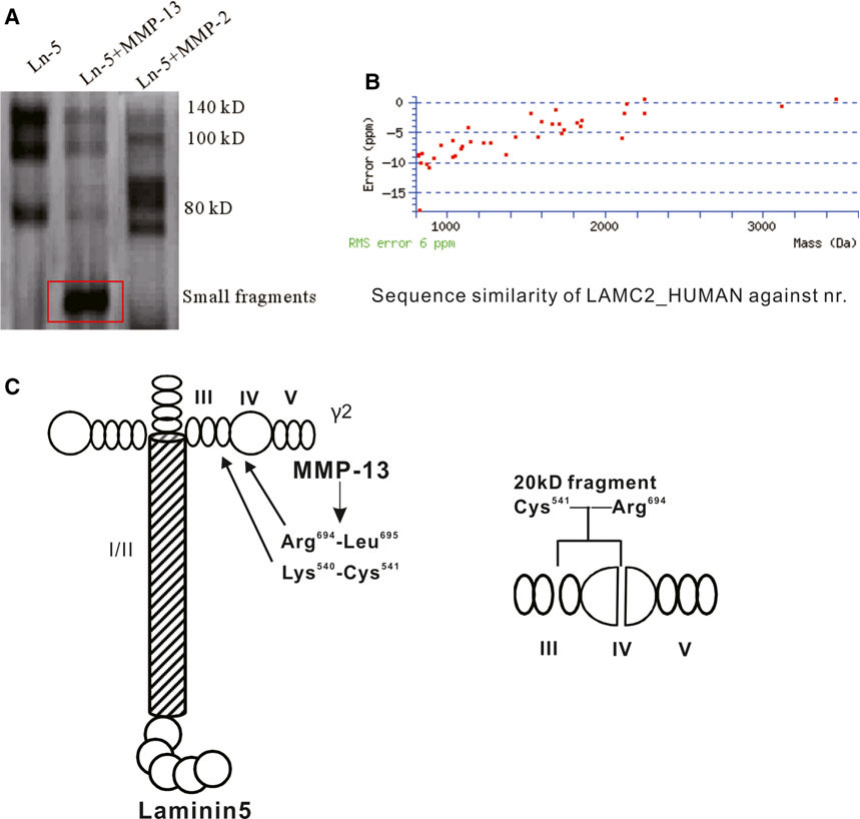
Cleavage of Ln‐5 by MMP‐13 was detected with a silver staining kit and LC‐MS/MS. (**A**) Human Ln‐5 was cleaved by MMP‐2 and MMP‐13 at 37°C for 6 hrs, and the cleavage fragments were separated on a 12% SDS‐PAGE gel and detected with a silver staining kit. The left lane shows the 140‐ and 100‐kDa forms of the intact Ln‐5γ2 chain. MMP‐2 generated an 80 kD Ln‐5γ2x fragment and a very faint 66 kD fragment. MMP‐13 further generated low molecular weight fragments that were approximately 20 kD. (**B**) LC‐MS/MS detection showed that the 20 kD fragment generated by MMP‐13 was part of the Ln‐5γ2 chain. (**C**) MMP‐13 cleaved the Ln‐5γ2 chain at Lys‐Cys (540–541) and Arg‐Leu (694–695). This fragment belongs to the Ln‐5γ2 chain III and IV domain.

We examined the MMP‐13 cleavage products of Ln‐5 and compared them with the MMP‐2 cleavage products of Ln‐5. The results show that MMP‐2 proteolytically cleaved Ln‐5 into the 105 kD Ln‐5γ2ʹ and 80 kD Ln‐5γ2x. However, MMP‐13 cleaved Ln‐5 into smaller fragments with molecular weights of approximately 20 kD (Fig. 2A). Using LC‐MS/MS, we obtained the amino acid sequence of the 20 kD fragment. The fragment contained 154 amino acids that are present in the Ln‐5γ2 peptide (Fig. 2B and C).

### The effect of MMP‐2 or MMP‐13 Ln‐5 cleavage fragments on EGFR and F‐actin expression

**Figure 3:**
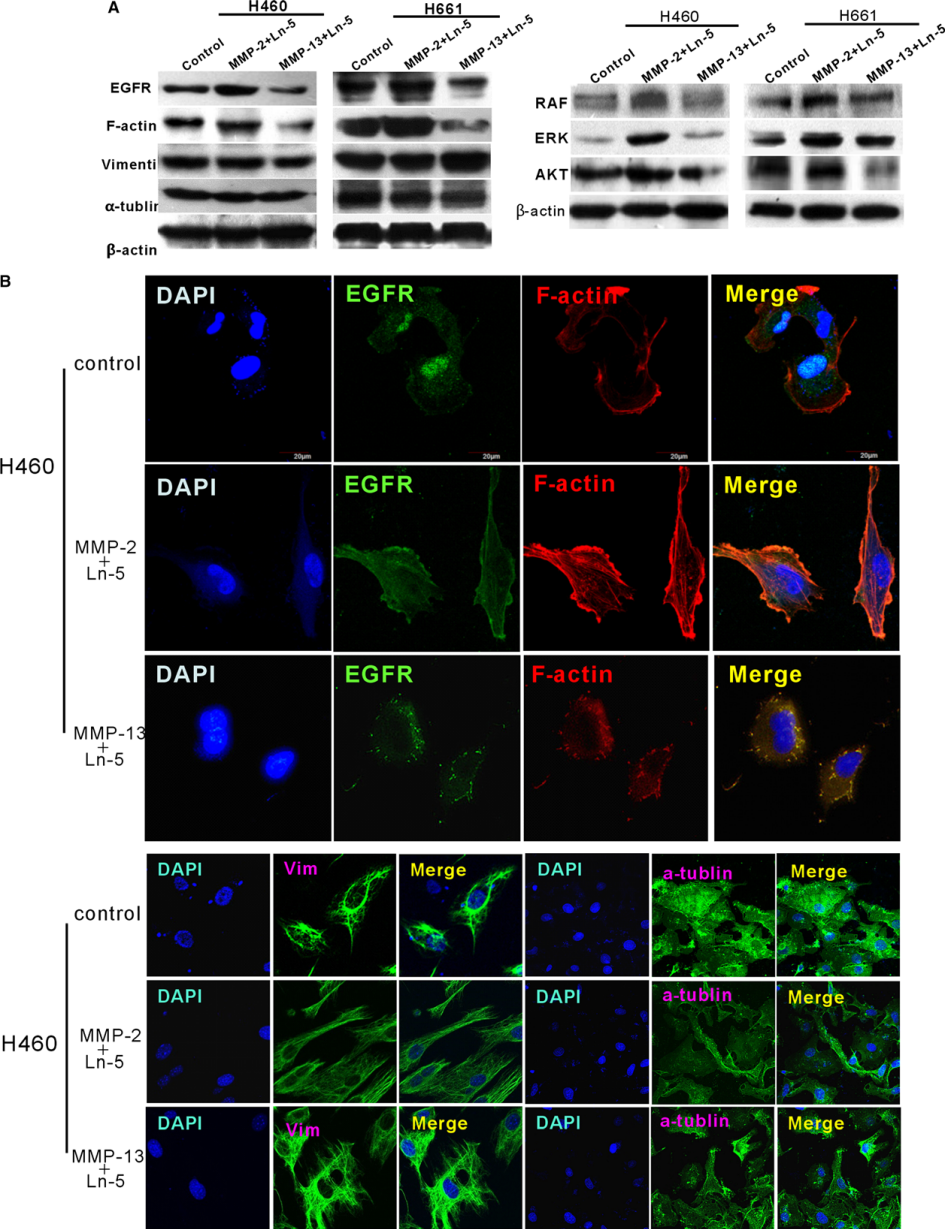
Cleavage of Ln‐5 by MMP‐2 or MMP‐13 affected EGFR and F‐actin expression. (**A**) The WB results show that treatment with MMP‐2 Ln‐5 cleavage fragments enhanced the EGFR, F‐actin and EGFR downstream targets Raf, ERK and AKT protein levels in cells, but the levels were reduced in the MMP‐13‐Ln‐5 group. Vimentin and α‐tubulin levels were not obviously changed in either group. (**B**) Immunofluorescence staining was performed, and higher co‐expression of EGFR and F‐actin filaments was observed in the MMP‐2 + Ln‐5 group, and the arrangement of the F‐actin cytoskeleton was dramatically changed compared with that of the MMP‐13 + Ln‐5 group. The expression of vimentin and α‐tubulin was not different between these two treatment groups in both H460 and H661 cells.

Epithelial tumour cells undergo conspicuous morphological changes during VM formation. Because Ln‐5γ2 contains an EGF domain, we hypothesized that the fragments resulting from MMP‐2 cleavage of Ln‐5 could promote VM formation by activating EGFR to induce cytoskeletal rearrangements, and MMP‐13 might destroy the EGF domain that induces changes in cellular morphology. The EGFR level, EGFR downstream targets Raf, ERK and AKT and the arrangement of F‐actin, vimentin and α‐tubulin in cells treated with different MMP‐2 or MMP‐13 cleavage fragments were analysed with WB and immunofluorescence staining assays. The WB results showed that treatment with MMP‐2 Ln‐5 cleavage fragments stimulated an increase in EGFR, Raf, ERK, AKT and F‐actin protein levels, whereas the expression of these proteins was reduced in the MMP‐13 + Ln‐5 group. WB demonstrated that the other Ln‐5 fragments had no effects on EGFR activation (Fig. S1B). Vimentin and α‐tubulin did not exhibit obvious changes in either group (Fig. 3A). Immunofluorescence staining showed increased co‐expression of EGFR and actin filaments in the MMP‐2 + Ln‐5 group, and the arrangement of the F‐actin cytoskeleton changed dramatically compared with that of the MMP‐13 + Ln‐5 group. The expression of vimentin and α‐tubulin was not changed between these two treatment groups in both H460 and H661 cells (Fig. 3B). These results indicate that MMP‐2 Ln‐5 cleavage fragments activate EGFR to stimulate the downstream signalling molecules, some of which participate in cytoskeleton reorganization and motility. MMP‐13 Ln‐5 cleavage fragments inhibit this activity.

### The expression of MMP‐13 and MMP‐2 in LCLC tissues

**Figure 4:**
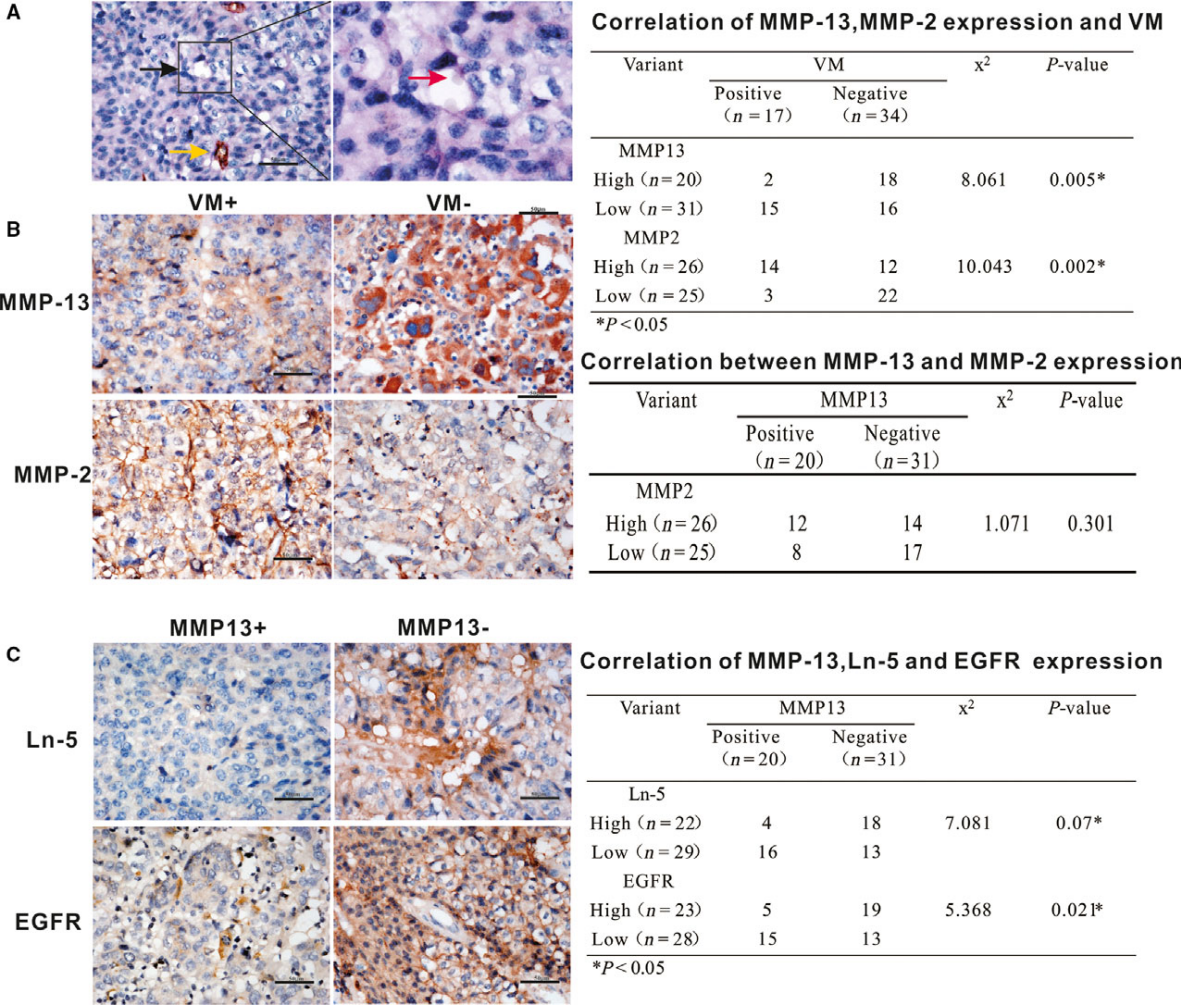
VM and the expression of MMP‐13, MMP‐2, Ln‐5 and EGFR in LCLC tissues. (**A**) Representative images of the presence of VM in human LCLC tissues (CD34/PAS double‐staining). The yellow arrow shows that the endothelial‐dependent vessel was positive for both CD34 and PAS. The black arrows show VM channels in which PAS‐positive materials and red blood cells (red arrow) are observed. (**B**) VM‐positive tissues exhibited significantly reduced MMP‐13 expression, and VM‐negative tissues exhibited significantly increased MMP‐13 expression (*P* = 0.005). In contrast, VM‐positive tissues exhibited significantly reduced MMP‐2 expression, and VM‐negative tissue exhibited significantly increased MMP‐2 expression. There was no significant correlation between MMP‐13 and MMP‐2 (*P* = 0.301). (**C**). Significantly reduced expression of Ln‐5 and EGFR can be observed in the LCLC samples with MMP‐13‐positive tissues compared with expression in VM‐negative tissues (*P* < 0.05) (Bar = 50 μm).

MMP‐13 has recently been shown to play a critical role in angiogenesis during fracture healing. To determine the relationship between MMP‐13 and VM, we examined the expression of MMP‐13 and its relationship with VM formation in clinical cancer cases. In 50 LCLC tissues, CD34/PAS double‐staining was used to identify VM (Fig. 4A). Positive expression of MMP‐13 was observed in 20 of 51 (39.2%) LCLC cases (Fig. 4B). As shown in the table in Figure 4B, the expression of MMP‐13 was negatively correlated with VM (*P* = 0.005). In contrast, a positive correlation was found between MMP‐2 and VM (*P* = 0.002). There was no significant correlation between MMP‐13 and MMP‐2 (*P* = 0.301). In addition, Ln‐5 and EGFR expression was negatively correlated with MMP‐13, and MMP‐13‐overexpressing tissues had low Ln‐5 and EGFR expression levels (Fig. 4C). These results confirm the *in vitro* experimental results that MMP‐13 can degrade Ln‐5 and inhibit EGFR activity.

### The effect of MMP‐13 on tumour growth and blood supply patterns *in vivo*

**Figure 5:**
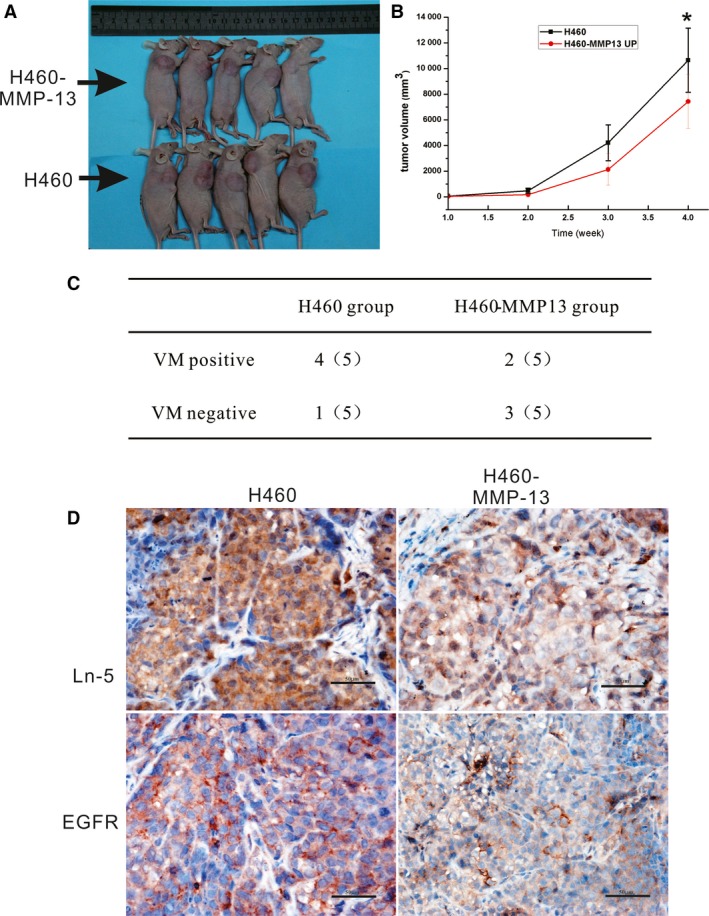
The effects of MMP‐13 transfection in xenografts. (**A**) Subcutaneous implantation of 5 × 10^6^ H460 cells or H460 pcDNA3‐MMP‐13‐transfected cells (H460‐MMP‐13) in BALB/c nude mice led to tumour formation. (**B**) BALB/c nude mice showing that 5 weeks after cell injection, xenografts in the MMP‐13‐transfection group developed more slowly than those in the H460 group (*P* < 0.05). (**C**) Xenografts in the H460‐MMP‐13 group showed attenuated VM formation ability. (**D**) Ln‐5, EGFR and VE‐cadherin were significantly down‐regulated in xenografts of the H460‐MMP‐13 group (Bar = 50 μm).

To further validate the effect of MMP‐13 on LCLC cells, we constructed xenograft mouse models. After the subcutaneous transplantation of H460 and H460‐MMP‐13‐overexpressing cells in nude mice, all 12 mice showed successful xenografts. Compared with the H460 group, we found that transplanted tumours in the H460‐MMP‐13‐overexpressing group developed more slowly. The volume of the xenografts was obviously different, and the tumour volume in the H460 group was larger than that in the H460‐MMP‐13 cells (10648.09 and 7435.60 mm^3^, respectively) (*P* < 0.05, Fig. 5A). VM was also examined in four of the mice in the H460‐transfection group and was found to be higher than that in the H460‐MMP‐13 group (2/5, *P* < 0.05, Fig. 5B). Moreover, compared with the control group, Ln‐5 and EGFR expression was decreased in the H460‐MMP‐13 group (Fig. 5C).

**Figure 6:**
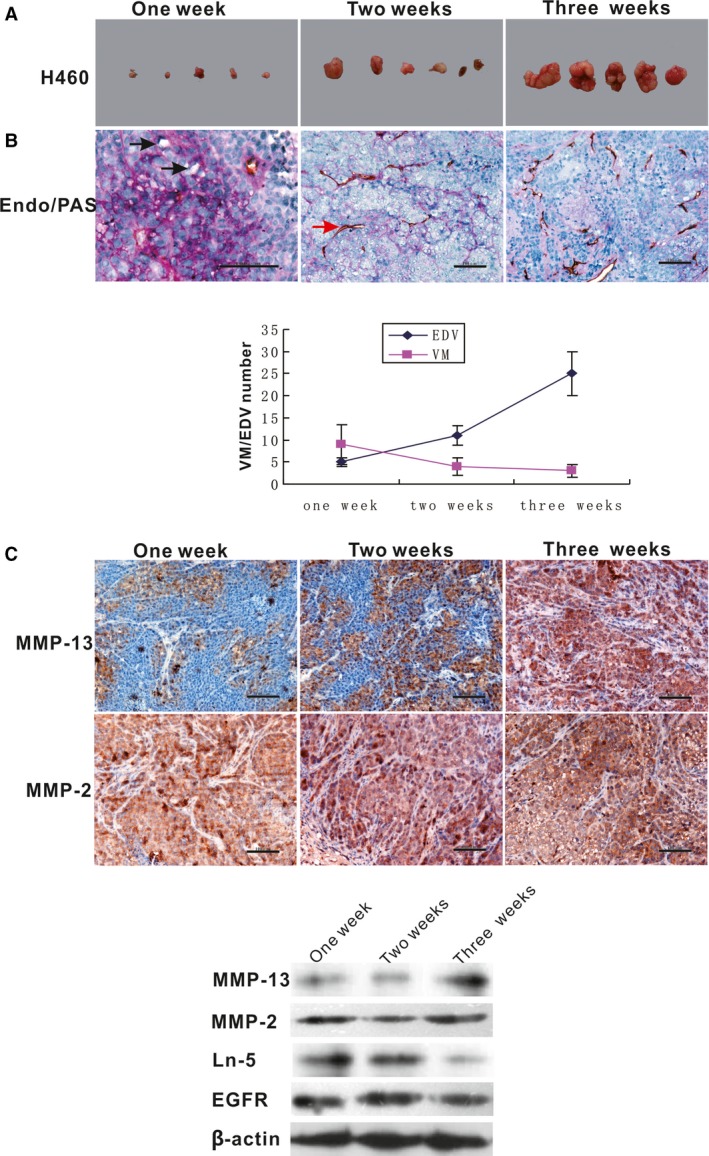
MMP‐13 and MMP‐2 expression in different tumour stages. (**A**) Tumour volume at different weeks. (**B**) Endomucin/PAS double‐staining in H460 xenografts showed decreased VM but increased EDV numbers (Bar = 100 μm). The black arrow shows VM formation. The red arrow shows the EDVs. (**C**) The expression levels of MMP‐13 showed a gradually increasing trend with tumour growth. Meanwhile, no significant difference in the MMP‐2 expression was observed at different tumour growth stages (Bar = 100 μm). WB showed MMP‐13 gradually increasing, but Ln‐5 and EGFR showed a decreasing trend with tumour growth. MMP‐2 expression was observed at different tumour growth stages.

To investigate the relationship between MMP‐13 and MMP‐2 expression and tumour blood supply patterns in different stages of LCLC, we examined the expression levels of MMP‐13 and MMP‐2 and tumour blood supply patterns in different stages of LCLC. The results confirmed the relationship between MMP‐13 expression and VM and EDVs, and a correlation analysis showed that MMP‐13 expression was negatively correlated with the number of VM networks in tumour cells but positively correlated with the number of EDVs (Fig. 6A and B). The expression levels of MMP‐13 showed a gradually increasing trend with tumour growth. WB showed that MMP13 was negatively correlated with Ln‐5 and EGFR expression in xenograft tissues. Meanwhile, we did not observe any significant differences in MMP‐2 expression, in contrast to MMP‐13 expression, which showed a time‐dependent increasing trend (Fig. 6C). MMP‐13 might play a role in promoting a VM to EDV transition.

## Discussion

This study demonstrated that Ln‐5 cleaved by MMP‐2 promoted VM formation by activating EGFR to enhance actin rearrangement. MMP‐13 cleaved Ln‐5 into small fragments, thereby abrogating the EGFR signal that disrupts the formation of VM patterns in lung cancer.

VM describes a process in which aggressive tumour cells in 3D matrices mimic embryonic vasculogenesis by forming ECM‐rich, patterned tubular networks. The highly aggressive cells can modify their ECM to initiate or promote the VM process. MMPs represent a family of zinc‐dependent proteinases, which degrade ECM components 14, 15. In our study, Ln‐5 fragments cleaved by MMP‐2 promoted VM formation, which is consistent with other studies, whereas Ln‐5 fragments cleaved by MMP‐13 were found to decrease VM formation. Laminins undergo proteolytic cleavages attributable to posttranslational (and possibly post‐secretion) modifications that result in laminin fragments, which may remain bound to the parent molecule 16 17. In this context, laminin degradation associated with basement membrane turnover and tissue remodelling can expose and render active specific laminin sequences that are capable of mediating cell interactions but that were buried and not functionally available before proteolytic degradation. We found that cells treated with Ln‐5 fragments cleaved by MMP‐2 showed increased EGFR expression and F‐actin cytoskeleton changes. The results confirmed that these small molecular fragments activated the EGFR pathway and may affect the cell cytoskeleton to promote VM formation. Ln‐5 fragments cleaved by MMP‐2 contain an EGF‐like domain, and these fragments may bind to tumour EGFRs to activate downstream signalling pathways. The EGFR pathway is involved in a variety of cellular behaviours and in crosstalk with several cell signalling pathways 18, especially in lung cancer, and EGFR has been used as an important factor for chemotherapy 19, 20. Cells undergoing VM were shown to have altered cytoskeletal structure with enhanced motility and less polarity. The associated motility signalling or functional molecules fascin, Arp3, vinculin and MLC, which play multiple roles in the assembly and arrangement of F‐actin in the activated cytoskeleton, are activated in downstream events and ultimately cause the cell to convert to an active state allowing for invasive motility. These cytoskeletal dynamics of migrating cells are strongly associated with the EGFR signalling pathway because there is intensive crosstalk between the cytoskeleton and EGFR signalling pathways 21, 22. Therefore, we propose that the motility‐cytoskeletal effects induced by the Ln‐5 fragments are likely due to the fact that the fragments function similarly to EGFR ligands to activate EGFRs and then evoke EGFR downstream signalling pathways to interrupt cytoskeleton construction and activate cell tube formation.

MMP‐13 plays an important role in the matrix degradation process, which has been confirmed to promote tumour invasion, migration and angiogenesis in a variety of tumours 23, 24, and thus, we initially believed it could promote the formation of VM. To our surprise, our results showed a negative correlation with VM formation. MMP‐13 overexpression or treatment with recombinant MMP‐13 hindered the ability of lung tumour cells to form tubular structures. In human LCLC tissue samples, we also found a negative correlation between MMP‐13 expression and tube formation. Our data showed that MMP‐13 could further proteolytically degrade the γ2ʹ fragments of Ln‐5 into smaller cleavage fragments, which is consistent with previous results. The mass spectrometry results showed that these small fragments did not contain a complete EGF‐like domain. We thus hypothesized that the disruptive effect of MMP‐13 on VM was possibly due to the destruction of the smaller Ln‐5 fragments cleaved by MMP‐2. The addition of these small fragments to the substrate in 3D cell culture did not decrease EGFR expression or F‐actin cytoskeleton changes. Therefore, we hypothesized that MMP‐13‐mediated Ln‐5 degradation undermines the functional domains of Ln‐5, thereby reducing its ability to promote VM formation. MMPs affect the biological behaviour of cells through ECM degradation 25, 26, and they also affect multiple signalling pathways that modulate the biology of cells during normal physiological processes and under disease‐related conditions 27, 28. Above all, we hypothesise that MMP‐13 possibly inhibits or disrupts VM formation in the following manner. MMP‐13 cleaves the Ln‐5 fragments Ln‐5γ2ʹ and Ln‐5γ2x into smaller fragments, which prevents the transfer of the EGFR molecular signal into the cell, preventing the signal from inducing the rearrangement of the actin cytoskeleton and affecting the vascular phenotype of aggressive tumour cells. Studies have shown that there are three microcirculation modes in tumours, which undergo dynamic exchanges in different stages of tumour development *via* the effects of cells and the microenvironment. VM is one of the microcirculation models in early tumour development. Although it plays an important role in the early stage of highly malignant tumour blood supply development, it is replaced by endothelium‐dependent vascular circulation, which is more suitable for providing adequate nutrition to tumours. In MMP‐13‐overexpressing xenografts, the number of VM channels was extremely low, and the tumour growth rate was significantly decreased. These data indirectly confirm the negative regulation of VM by MMP‐13. Our observations in the xenograft mouse model further showed a decrease in VM and an increase in endothelium‐dependent vascularization with the gradual increase in tumour volume. In this process, there was no significant change in the expression of MMP‐2, but the expression of MMP‐13 gradually increased. This suggests that MMP‐13 may play a major role in regulating tumour blood supply in the late stages of tumour growth. In other words, MMP‐2 may play a catalytic role in the formation of VM, which is a fast and efficient response mechanism to provide blood to the tumour and provides the major blood supply in the early stage. With tumour growth, certain factors, such as persistent tumour hypoxia‐mediated activation of HIF‐2α 29, may promote transcription and high expression levels of MMP‐13 30, which plays a role in inhibiting the formation of VM through Ln‐5 degradation. MMP‐13 degrades ECM components, including Ln‐5, continuously promoting endothelial cell migration in the ECM, the release of VEGF and bFGF, and ultimately the generation of endothelium‐dependent vascular tumours. Then, the vascular network of the tumour is established and continues to maintain tumour growth, invasion and metastasis.

Although further research is needed, the results of this study indicate that MMP‐13 may be used as a therapeutic target to inhibit blood supply in combination with anti‐EDV growth drugs in LCLC. Given that MMP‐13‐specific inhibitors have already been developed, this study supports the evaluation of these inhibitors for the treatment of LCLC.

## Conflicts of interest

The authors declare no conflicts of interest.

## Supporting information


**Figure S1** The effects of different MMP‐2 and MMP‐13 Ln‐5 cleavage fragments on EGFR activation and VM formation.
