# Supplementary figures and images for: MMP‐2 and MMP‐13 affect vasculogenic mimicry formation in large cell lung cancer
Source: J Cell Mol Med. 2017 Aug 2;21(12):3741–51. doi: 10.1111/jcmm.13283 (PMC5706571; doi:10.1111/jcmm.13283)

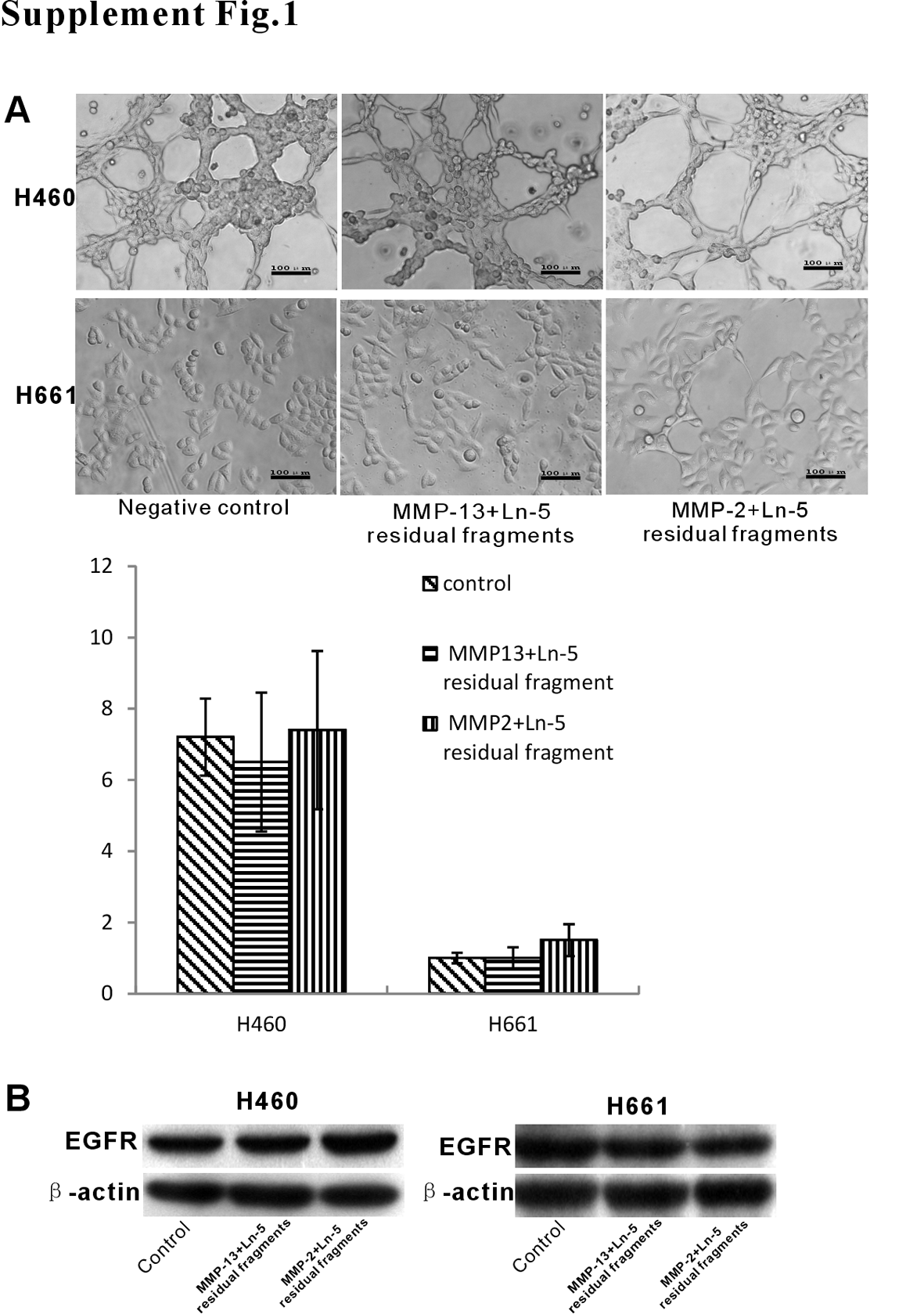

Supplement: Supplementary file 1 — Figure S1 The effects of different MMP‐2 and MMP‐13 Ln‐5 cleavage fragments on EGFR activation and VM formation. [file JCMM-21-3741-s001.tif]
